# Chloroplast: The Emerging Battlefield in Plant–Microbe Interactions

**DOI:** 10.3389/fpls.2021.637853

**Published:** 2021-03-03

**Authors:** Feng Yang, Kunqin Xiao, Hongyu Pan, Jinliang Liu

**Affiliations:** College of Plant Sciences, Jilin University, Changchun, China

**Keywords:** chloroplast immunity, phytohormone, light-harvesting complex, CAS, retrograde signaling pathway, effectors

## Abstract

Higher plants and some algae convert the absorbed light into chemical energy through one of the most important organelles, chloroplast, for photosynthesis and store it in the form of organic compounds to supply their life activities. However, more and more studies have shown that the role of chloroplasts is more than a factory for photosynthesis. In the process of light conversion to chemical energy, any damage to the components of chloroplast may affect the photosynthesis efficiency and promote the production of by-products, reactive oxygen species, that are mainly produced in the chloroplasts. Substantial evidence show that chloroplasts are also involved in the battle of plants and microbes. Chloroplasts are important in integrating a variety of external environmental stimuli and regulate plant immune responses by transmitting signals to the nucleus and other cell compartments through retrograde signaling pathways. Besides, chloroplasts can also regulate the biosynthesis and signal transduction of phytohormones, including salicylic acid and jasmonic acid, to affect the interaction between the plants and microbes. Since chloroplasts play such an important role in plant immunity, correspondingly, chloroplasts have become the target of pathogens. Different microbial pathogens target the chloroplast and affect its functions to promote their colonization in the host plants.

## Introduction

Chloroplasts are considered to be organelles produced by endosymbiotic bacteria in plants that undergo photoautotrophy (Ding et al., 2019). Through coexistence and evolution in billions of years, the photosynthetic cyanobacteria, then chloroplasts, become indispensable parts of eukaryotic plant cells (Gray, 1989; Cavalier-Smith, 2002; Ziehe et al., 2017). During the coevolution process with host cells, most of the prokaryotic genomic contents of cyanobacteria are transferred to the host cell nucleus, resulting in approximately 100 genes in the current chloroplast genome (McFadden, 2001; Martin et al., 2002; Daniell et al., 2016). Most of the chloroplast-targeted proteins encoded in host nucleus contain a chloroplast transit peptide (cTP) at their N-terminal, which subsequently enters chloroplasts by interacting with the translocators located in the inner membrane (TIC) and outer membrane (TOC) of the chloroplast and could be cleaved off by a stromal processing peptidase (SPP) (Bruce, 2000; Jarvis and Soll, 2001; Day and Theg, 2018; Richardson et al., 2018). Some chloroplast-targeted proteins do not contain a cleavable N-terminal cTP, while they enter chloroplasts through some non-classical ways (Jarvis and Robinson, 2004; Richly and Leister, 2004; Beck, 2005; Bedard, 2005; Beale, 2011), and the examples include substrate-dependent import of pPORA proteins (Reinbothe et al., 2000). In addition, the *Arabidopsis* chloroplast heat shock proteins, AtcpHsp70-1 and AtcpHsp70-2, contain age-selective signals in transit peptides, which enable them to preferentially enter chloroplasts in mature stage (Teng et al., 2012).

Chloroplast is the organelle containing chlorophyll for photosynthesis and is the main source of reactive oxygen species (ROS), which can lead to a damage of the photosystem II (PSII) reaction center (Krieger-Liszkay et al., 2011). Overreduction of PSII and oxygen molecules by excessive illumination leads to the formation of ROS, including singlet oxygen (^1^O_2_) and superoxide anion radicals (O_2_$\bar{\cdot }$), and O_2_$\bar{\cdot }$ dismutates to form free H_2_O_2_ that is then reduced to hydroxyl radical (HO⋅) (Pospíšil et al., 2004). Overaccumulation of ROS could damage the plant growth and development. Meanwhile, the plant-defense-related hormones, salicylic acid (SA), and jasmonic acid (JA), for example, are synthesized in the chloroplasts.

Unlike animals, plants cannot move to escape the invasions from natural enemies, which consequently have evolved a complex multilayered immune system to protect themselves from microbial infection and insect feeding. Plasma membrane (PM)-localized pattern-recognition receptors (PRRs) consist of the first layer of immune system, which perceive the pathogen-associated molecular pattern (PAMP) and trigger immunity [PAMP-triggered immunity (PTI)]. On the other hand, certain specific effectors can be directly or indirectly recognized by plant intracellular nucleotide-binding leucine-rich repeat domain-containing receptor (NLR), second layer of the immune system, to trigger a robust immune response [effector-triggered immunity (ETI)] (Jones and Dangl, 2006; Zipfel, 2014; Liu C. et al., 2019). Once the presence of microbes has been perceived by plants, the signaling cascades are then activated, which in turn initiate a multilayered immune response, and the outputs of this combat, resistance or susceptibility, between plant and pathogens depends on several factors, in which chloroplasts play significant roles.

## From Invasion Signal Perception to Chiloroplast

PAMP-triggered immunity and ETI immune responses vary largely in the magnitude and duration but result in similar downstream molecular events, such as mitogen-activated protein kinase (MAPK) activation, oxidative burst, ion influx, Ca^2+^ signaling, increased biosynthesis of plant defense hormones, and transcriptional reprogramming (Peng et al., 2018). After PAMP perception, several lines of evidence demonstrate that PTI response triggers Ca^2+^ influx at the plasma membrane (Ranf et al., 2011; Nomura et al., 2012). Although several proteins were suggested to be potential Ca^2+^ channels in plants, for example, ionotropic glutamate receptor-like channels (GLRs), cyclic nucleotide gated channels (CNGCs), and mechanosensitive MCA-like channels (MCAs), one of the most recent works indicate that CNGC2 and CNGC4 in *Arabidopsis* are essential for PAMP-induced Ca^2+^ signaling, which constitute a functional channel and is phosphorylated by receptor-like cytoplasmic kinase BIK1, a core PTI regulator, to increase the cytosolic calcium concentration (Tian et al., 2019). A similar finding in rice suggests that OsCNGC9 is phosphorylated by OsRLCK185 after PAMP treatment to activate the calcium channel (Wang et al., 2019), supporting the model that RLCK-mediated phosphorylation of CNGCs plays important roles in PTI. The identity of plasma membrane Ca^2+^ channels responsible for the Ca^2+^ influx in ETI is still unknown (Seybold et al., 2014), although many Ca^2+^-responsive proteins have been identified as critical regulators of plant immunity.

Calcium-sensing receptor (CAS) is a calcium-binding protein located on the thylakoid membrane in the chloroplast. Once PTI or ETI signal is relayed to the chloroplast, Ca^2+^ from the thylakoid lumen, which contains a high concentration of Ca^2+^, are transported to the stroma by CAS, resulting in a continuous high concentration of Ca^2+^ in the stroma, and the signal is then transduced from the chloroplast to the nucleus through the ^1^O_2_-mediated retrograde signaling pathway (Kim and Apel, 2013), which regulates the defense responses through transcriptional reprogramming of defense-related genes (Nomura et al., 2012). Another outstanding example is that the calcium protein kinase 16 (CPK16), localized on the PM, undergoes N-myristoylation in normal condition and relocalizes from the PM to chloroplasts upon flg22 or immune elicitors treatment to promote chloroplast-dependent defenses (Medina-Puche et al., 2020).

Both PTI and ETI responses activate MAPK pathway in a short and sustained manner. The sustained MPK3 and MPK6 activation, triggered by ETI response, could inhibit photosynthesis, decrease the CO_2_ fixation, increase the ROS accumulation and the programmed cell death (PCD), and increase the synthesis of defense-related secondary metabolites (Su et al., 2018). Once plants sense invasions, they can actively inhibit photosynthesis and thus allocate more energy to the immune responses (Nomura et al., 2012). On the other hand, pathogens could decrease the photosynthesis in plants by secreting metabolites and proteins (Rodríguez-Herva et al., 2012; Bhattacharyya et al., 2015; Schmid et al., 2016; Xu et al., 2019), which directly affect the accumulation of ROS (Rodríguez-Herva et al., 2012; Zhou et al., 2015; Xu et al., 2019). ROS are considered to play different roles in combating different pathogens, which promote the infection of necrotrophs while inhibiting biotrophs, since ROS is not only an important signaling molecule but also a toxic factor to cells that is often related to hypersensitive response (HR) and programmed cell death (PCD) (Govrin and Levine, 2000; Glazebrook, 2005).

Guanosine tetraphosphate [(p)ppGpp] is a regulator of chloroplast gene expression. Plants with excessive accumulation of (p)ppGpp show defects in chloroplast function, with upregulation of large amounts of chloroplast function-related genes, while plants with low (p)ppGpp levels show increased SA accumulation, premature expression of PR genes, and increased resistance to *Turnip mosaic virus* (TuMV) infection (Abdelkefi et al., 2018).

## Immune Responses Inside Chloroplast

The light-harvesting complex II (LHCII) surrounds PSII to absorb light and transmit to PSII (Sheng et al., 2018), in which the light is converted into chemical energy. *Magnaporthe oryzae*, a filamentous fungal pathogen, causes devastating rice blast disease on rice, and the light-induced phosphorylation of light-harvesting complex II protein (LHCB5) enhances broad-spectrum resistance of rice to *M. oryzae* (Liu M. et al., 2019). The members of the LHCB family are important in photosynthesis and guard cell signaling in response to abscisic acid (ABA), while downregulation or disruption of LHCB leads to less sensitive to ABA and ABA-regulated stomatal movement in guard cells and partly destroys ROS homeostasis (Xu et al., 2012). ABA-mediated stomata closure can effectively prevent the invasion of microbes (Lim et al., 2015).

Intracellular ROS are mainly produced in the chloroplasts, which can act as signaling molecules to affect localized cell death (LCD) in non-host resistance. The non-host resistance 2 (NHR2) protein of tobacco and *Arabidopsis* was proposed to act as a new component of the chloroplast-signaling pathway to activate the callose deposition to the cell wall in response to bacterial pathogens and enhance non-host disease resistance (Singh et al., 2018). Tobacco overexpressing flavodoxin (Fld) accumulates less ROS in chloroplasts, which leads to the inhibition of LCD after inoculation of the non-adapted bacterium *Xanthomonas campestris* pv. *vesicatoria* (Pierella Karlusich et al., 2017). In addition, studies have shown that overexpression of plastid-targeted cyanobacterial flavodoxin (pfld) in tobacco results in significantly reduced accumulation of ROS in chloroplast, which in turn leads to the enhanced resistance to *Botrytis cinerea*, suggesting that ROS derived from chloroplast plays an important role in the resistance of plants to necrotrophic fungus (Rossi et al., 2017). The h-type thioredoxin TRXh3 in tobacco is specifically located in the chloroplast and maintains the reduced state of cells. Tobacco plants overexpressing TRXh3 show increased resistance to *Tobacco mosaic virus* (TMV) and *Cucumber mosaic virus* (CMV) (Sun et al., 2010).

## The Chloroplast: Factory for JA and SA Production

The plant defense responses are based on a highly regulated and complex network of phytohormone signaling pathways, in which SA and JA are thought to be the backbone (Li et al., 2019). Chloroplasts can regulate the production of SA and JA. The SA-mediated defense response restricts the spread of biotrophic and hemibiotrophic pathogens, while the JA-mediated defense response mainly targets the necrotrophic pathogens. It is generally agreed that the SA- and JA-mediated signaling pathways are mutually antagonistic. This notion is well supported by the case of a bacterial pathogen *Pseudomonas syringae* that secretes a phytotoxin coronatine (COR), mimicking the jasmonate-isoleuce (JA-Ile) to promote the degradation of the Jasmonate Zim domain (JAZ) protein and to activate the JA-induced defense, thus inhibiting the SA-mediated defense (Bender et al., 1998, 1999). Different from bacteria, in which isochorismate is directly converted to SA by an isochorismate pyruvate lyase, SA in plants is synthesized through the phenylalanine ammonia-lyase (PAL) and isochorismate synthase (ICS) pathways (Dempsey et al., 2011). Moreover, the accumulation of SA caused by pathogen invasion is mainly through ICS pathway. Chorismate, the product of the shikimate pathway, is catalyzed by ICS to form isochorismate in the chloroplast, which is then transported to cytoplasm by enhanced disease susceptibility 5 (EDS5) (Rekhter et al., 2019), and is then catalyzed by avrPphB Susceptible 3 (PBS3) to form IC-9-Glu, and is finally converted to SA spontaneously or catalyzed by enhanced pseudomonas susceptibility 1 (EPS1) (Torrens-Spence et al., 2019). Accumulation of SA was shown to be essential for systemic acquired resistance (SAR) (Gaffney et al., 1993). In the infected leaves, the syntheses of SA and pipecolic acid (Pip) are enhanced. Then, Pip and/or its derivative N-pipecolic acid (NHP) moves through the phloem to the distal uninfected leaves (Chen et al., 2018), increase the stability of non-expresser of PR genes 1 (NPR1) protein, which then activates SA and Pip biosynthesis and SAR at low SA concentration (Kim et al., 2020; Sun et al., 2020). The precursor of JA biosynthesis (9S, 13S)-12-oxo-phytodienoic acid (OPDA) is synthesized in chloroplasts. The galactolipids on the chloroplast membranes are catalyzed by fatty acid desaturase (FAD) and phospholipase A1 (PLA1) to release α-linolenic acid (α-LeA/18:3) (Wasternack and Song, 2017), which then synthesizes OPDA under the actions of 13-lipoxygenase (LOX), allene oxide synthase (AOS), and allene oxide cyclase (AOC); then, OPDA is transferred to the peroxisome to synthesize JA (Huang H. et al., 2017). In the process of ABA biosynthesis, the synthesis of carotenoid precursor and the formation and cleavage of xanthophyll all occur in plastids (Seo and Koshiba, 2002).

## Stromules and Chloroplast Movement

As a multicellular organism, communication between organelles is crucial for plant immune responses (Théry et al., 2009). Chloroplasts in tobacco epidermal cells are mainly located on the outer edge of the cell due to the extrusion of the huge central vacuole. Once the defense responses are activated, the chloroplasts quickly rearrange, cluster around the nucleus, and establish a connection with the nucleus through sending out dynamic tubular elongation, called stromules (Natesan et al., 2005). The stromule is a dynamic structure that depends on microtubules (MTs) and actin filaments (AFs). The top of the stromule interacts dynamically with MT and extends along the MT, which maintains their length by repeating the process of elongation and contraction. Although AFs are not required for the extension of stromule, they provide an anchor point. The stromules guide or push the chloroplast toward the nucleus by tightly binding the AFs around the nucleus, facilitating the relocalization of chloroplasts to nuclei during innate immunity (Kumar et al., 2018). The stromules connect the plastids and exchange the metabolites in the stroma (Hanson and Hines, 2018). N receptor interacting protein 1 (NRIP1), which localizes in the chloroplast normally, is recruited to the cytoplasm and nucleus during plant immunity (Caplan et al., 2008). Chloroplasts may transmit defense-promoting signals (such as NRIP1, H_2_O_2_) to the nucleus or other subcellular compartments through the stromules, and the formation of stromules precedes HR-PCD and pro-PCD signals (Serrano et al., 2016). Defense-related molecules H_2_O_2_ and SA can induce the formation of stromules. Since the pro-defense signals originate from the chloroplast, the increased surface area caused by the stromules may help their transfer to the cytoplasm and nucleus (Caplan et al., 2015). Ferredoxin2 is distributed in the chloroplasts clustered around the stromules and nucleus, indicating that it is likely to be transmitted from the chloroplast to the nucleus through the stromules (Wang et al., 2018). The accumulation of chloroplasts around the nucleus is a common response of plants upon activation of immune responses. In *Nicotiana benthamiana*, activation of PTI or ETI, transient expression of the replication-associated protein (Rep) from three different geminiviruses, or infection with RNA viruses or phytopathogenic bacteria can trigger this response (Kwok and Hanson, 2004; Caplan et al., 2008), which also occurs in non-infected cells. Therefore, it is very likely that a certain signal molecule produced by the chloroplast that is clustering around the nucleus can act as a system signal to transmit information, probably related to defense response, to eventually neighboring cells. Exogenous application of H_2_O_2_ is sufficient to induce the chloroplast perinuclear clustering, and the inhibition of intracellular ROS production reduces the number of clustered chloroplasts, indicating that ROS is necessary to induce the relocalization of chloroplasts. Therefore, this response is likely to be produced by sensing and accumulating ROS (Ding et al., 2019). In addition, the chloroplasts can also navigate toward the pathogen interface. During the infection of *Phytophthora infestans*, the *N. benthamiana* chloroplasts can navigate to the pathogen interface. Chloroplast unusual positioning 1 (CHUP1) is necessary for the repositioning of chloroplast by regulating the anchoring of chloroplast to the plant-derived extra-haustorial membrane (EHM). Chloroplasts can be recruited to the interface where the pathogen infection structure haustoria presents and establish contact with EHM by forming stromules. The plant cells may generate mechanical pressure through chloroplasts cluster and destroy the infection structure of pathogens that invade plant cells. However, *P. infestans* can inhibit the formation of stromules by secreting the effector AVR3a and reduce the contact area with chloroplast (Toufexi et al., 2019). Plant viruses can utilize endogenous host transport machinery to facilitate their intracellular movement (Laporte et al., 2003; Genovés et al., 2010). The AFs play an important role in the formation of stromules, which may be involved in the cell-to-cell transport of the virus in the host (Bhattacharyya and Chakraborty, 2018).

## Communication Between Chlorplast and Cell Nucleus

The chloroplasts are important environmental sensors and signaling hubs that transmit the developmental and environmental signals to the nucleus, thus regulating the expression of thousands of nucleus-encoded genes, called retrograde signaling (Chan et al., 2016). The SAL1-PAP retrograde signaling pathway mutant *sal1* shows more severe symptoms after infection with either the hemibiotrophic pathogen *P. syringae* pv. *tomato* DC3000 or the necrotrophic pathogen *Pectobacterium carotovorum* subsp. *carotovorum* EC1, and the ETI responses and the signaling pathways-mediated by SA and JA are attenuated (Ishiga et al., 2017). The metabolite of the plastidial retrograde signaling pathway, methylerythritol cyclodiphosphate (MEcPP), can increase the abundance of the red light receptor phytochrome B protein, then reduce the level and distribution of auxin as well as the content of ethylene, to adjust plant growth. MEcPP mediates the coordination of light and hormone signaling cascades, reprogramming plant growth in response to light (Jiang et al., 2020). The stress-responsive GCN2 kinase phosphorylates the translation initiation factor eIF2α to control cytosolic messenger RNA (mRNA) translation globally, while the ROS produced by chloroplasts can rapidly activate this process to regulate plant growth under stresses (Lokdarshi et al., 2020). ^1^O_2_, one of ROS produced by plant cells, can not only cause photodamage of the photosynthetic apparatus but also is related to the retrograde signaling pathways (Asada, 2006; Dogra et al., 2018). The ^1^O_2_-mediated oxidative posttranslational modification of the ^1^O_2_ sensor EXECUTER1 (EX1) is necessary to activate the ^1^O_2_-triggered retrograde signaling pathway (Dogra et al., 2019). The metalloprotease FtsH2 coordinates the retrograde signaling pathway triggered by ^1^O_2_ through the proteolysis of EX1 protein (Wang et al., 2016; Dogra et al., 2017).

## Chloroplast Are Targeted by Pathogens

Some microbes secrete proteins, named effectors, into plant cells, and certain effectors could enter chloroplasts and target the chloroplast-localized proteins (summarized in Table 1) owing to the leading sequences similar to the host chloroplast transit peptide. Pst_12806 secreted by *Puccinia striiformis* f. sp. *tritici* (Xu et al., 2019) interacts with the wheat TaISP protein in chloroplasts (Zheng et al., 2013). ISP protein is a subunit of cytochrome b6-f complex and has a C-terminal Rieske domain, which is responsible for the electron transfer (Yamori et al., 2011). Pst_12806 may weaken the electron transfer ability of the Rieske domain by interacting with the C-terminal of TaISP protein, then inhibits photosynthesis and the by-products ROS production, thereby inhibiting the host cell death and promoting pathogen survival (Xu et al., 2019). Chloroplast-targeted protein 1 (CTP1) secreted by *Melampsora larici-populina* (Petre et al., 2016) and the secreted protein PvRXLR8620 of *Plasmopara viticola* (Liu et al., 2018) can accumulate in the chloroplasts. Nuclear-encoded chloroplast protein glycerate 3-kinase (GLYK) is a basal immunity-related kinase in plants, while the plant pathogen *P. infestans* attempts to interfere with its function by secreting the effector AVRvnt1 (Pais et al., 2018). AVRvnt1 interacts with GLYK and promotes its degradation by the proteasome to prevent it from translocating to the chloroplast. However, the plant NLR protein Rpi-vnt1.1 monitors the transport of GLYK, recognizes AVRvnt1, and triggers immune responses. The light-dependent alternative promoter selection (APS) causes the plant to produce a truncated GLYK under dark conditions, which cannot interact with AVRvnt1 (Gao et al., 2020). SsITL, an integrin-like protein, is secreted by fungal pathogen *Sclerotinia sclerotiorum*, which suppresses host immune response at the early stage of infection by binding to the *Arabidopsis* CAS in the chloroplast and inhibiting the accumulation of SA (Zhu et al., 2013; Tang et al., 2020). The early defense response activated by PAMP causes a rapid decrease in non-photochemical quenching (NPQ) (Göhre et al., 2012). However, the oxalic acid secreted by *S. sclerotiorum* acidifies the infected tissue of *A. thaliana* during infection, which limits the protons flux into the stroma, downregulates the activity of ATP synthase, protonizes the PsbS protein, and activates the violaxanthin de-epoxidase (VDE) enzyme to increase NPQ. In addition, violaxanthin, as the precursor of the xanthophyll cycle, plays an important role in the synthesis of the plant hormone ABA (Pasqualini et al., 1999). The activated VDE enzyme catalyzes the conversion from violaxanthin to zeaxanthin, limiting the biosynthesis of ABA, and ultimately affects the plant defense responses, including the production of ROS and callose deposition (Zhou et al., 2015). *Rhizoctonia solani* causes host chloroplast deformities, weakens the photosynthesis, and perturbs plant hormone signaling to promote the infection (Ghosh et al., 2017). The endophytes in *Lolium perenne* systemically but moderately increases the hormones biosynthesis and weakens the photosynthesis to increase its resilience (Schmid et al., 2016).

**TABLE 1:** Microbial proteins target plant chloroplasts.

Microbes	Protein name	Annotation	Implication of interaction	References
*Alfalfa mosaic virus*	CP	Coat protein	Segregate the chloroplast protein PsbP	Balasubramaniam et al., 2014
*Sclerotinia sclerotiorum*	ITL	Integrin-like protein	Binding CAS and inhibiting SA synthesis	Tang et al., 2020
*Puccinia striiformis*	Pst_12806	Haustorium-specific protein	Interacts with TaISP	Xu et al., 2019
*Phytophthora infestans*	AVRvnt1	RxLR type of effector protein	Interacts with and destabilizes GLYK	Gao et al., 2020
*Pseudomonas syringae*	Hopl1	Previously named HopPmaI	Causes chloroplast thylakoid structure remodeling and suppresses SA accumulation	Jelenska et al., 2007
*Pseudomonas syringae*	HopN1	Cysteine protease	Degraded PsbQ and inhibited PSII activity	Rodríguez-Herva et al., 2012
*potato leafroll virus*	CP	Capsid readthrough domain	Form an extensive interaction network with chloroplast-localized proteins	Deblasio et al., 2018
*Tomato yellow leaf curl virus*	C4		Inhibit the intercellular spread of RNAi through interact with BAM1	Rosas-Diaz et al., 2018
*Bamboo mosaic virus*	Rep	Replicase	Interacts with NbcpHsp70-2	Huang Y. W. et al., 2017
*Pseudomonas syringae*	HopZ1a	HopZ effector family of cysteine-proteases	Suppresses SA and EDS1-dependent resistance.	Macho et al., 2010
*Pseudomonas syringae*	HopBB1	Type III effector	Activates TCP14-repressed JA response genes	Yang et al., 2017
*Pseudomonas syringae*	HopX1	Cysteine protease	Interacts with and promotes the degradation of JAZ proteins	Gimenez-Ibanez et al., 2014
*Ralstonia solanacearum*	RipAL	Contains a putative lipase domain	Induces jasmonic acid production to suppress salicylic acid-mediated defense responses	Nakano and Mukaihara, 2018
*Candidatus* Liberibacter asiaticus	SahA	Salicylic acid hydroxylase	Degrade SA and suppress plant defenses	Li et al., 2017
*Tomato yellow leaf curl virus*	C4	C4 Protein	Suppression of SA responses	Medina-Puche et al., 2020
*Phytophthora infestans*	AVR3a	Host-translocated effector	Perturb pathogen induced stromule development	Toufexi et al., 2019
betasatellite	βC1	A suppressor of gene silencing	Destroy chloroplast ultrastructure and interact with PsbP to hampers non-specific binding of PsbP to the geminivirus DNA	Bhattacharyya et al., 2015; Gnanasekaran et al., 2019

*Candidatus* Liberibacter asiaticus (*C*Las) promotes the production of H_2_O_2_ by targeting host chloroplast and inhibits the expression of host antioxidant enzyme genes influencing the detoxification of the host. In addition, through upregulating the expression of ATP synthase, *C*Las induces the production of ATP in plant cells and then steals ATP to feed itself (Pitino et al., 2017). *C*Las secretes effector Las5315 (mature protein) to target the chloroplast of the host. In tobacco, it induces cell death and strong callose deposition, which is the main cause for the phloem plugging (Pitino et al., 2016). Moreover, *C*Las injects an SA hydroxylase SahA to suppress plant defense responses by degrading plant endogenous SA and inhibiting the expression of PR genes, which is similar to the observations in many other bacteria that encode the SA hydroxylases as well (Li et al., 2017). Some SA analogs, due to the structural difference, cannot be degraded by SA hydroxylase; therefore, they can neutralize the effect and effectively control the disease (Palmer et al., 2019). *P. syringae* delivers effectors into the host cell through the type III secretion system (T3SS) and disrupts the PSII by reprogramming the nuclear-encoded chloroplast-targeted genes (NECGs) (De Torres Zabala et al., 2015). HopK1, secreted by *P. syringae*, is a well-studied example that localizes in the chloroplast and can suppress the hypersensitive response (Li et al., 2014). Another *P. syringae* effector HopI1, entering the chloroplasts through a non-canonical pathway, although containing a chloroplast transit peptide sequence, can remodel chloroplast thylakoid structure and inhibit SA-mediated defenses (Jelenska et al., 2007). In addition, HopI1 interacts with the host heat shock protein Hsp70 through its C-terminal J domain, promoting pathogenesis by affecting Hsp70 activity (Jelenska et al., 2010). The cysteine protease HopN1, secreted by *P. syringae*, also locates in the chloroplasts and inhibits host immunity by degrading PsbQ protein and inhibiting PSII activity in tomato (Rodríguez-Herva et al., 2012). Some other effectors, for example, HopZ1a, HopBB1, and HopX1, secreted by *P. syringae*, can activate JA signaling pathway through targeting the JA repressor JAZ (Macho et al., 2009, 2010; Jiang et al., 2013; Gimenez-Ibanez et al., 2014; Yang et al., 2017). *Ralstonia solanacearum* secretes effector RipAL, which contains a putative lipase domain and targets the chloroplast lipids in plant cell, to promote JA biosynthesis by catalyzing the release of linoleic acid, an important precursor for JA biosynthesis, and succeeds in infection (Nakano and Mukaihara, 2018).

The geminivirus betasatellite that causes radish curl leaf disease (RaLCB) affects the ultrastructure and function of the host chloroplast by secreting βC1 protein to promote disease (Bhattacharyya et al., 2015). Tobacco PsbP protein can bind to geminivirus DNA and activate the defense response against the virus, while βC1 interacts with PsbP to prevent its non-specific binding to geminivirus DNA (Gnanasekaran et al., 2019). The *Alfalfa mosaic virus* (AMV) coat protein (CP) interacts with the *Arabidopsis* chloroplast-targeted PsbP protein in the cytoplasm, and overexpression of PsbP greatly reduces the virus accumulation in the infected leaves, suggesting that AMV may use this method to sequestrate PsbP protein to control the induced host resistance (Balasubramaniam et al., 2014). Viruses can promote their replication and movement through target host components (Maule et al., 2002; Pallas and García, 2011; Gray et al., 2014). The chloroplast-localized RNA helicase increased size exclusion limit 2 (ISE2) can regulate the formation and distribution of plasmodesmata. Virus infection can induce the expression of ISE2 in the host. In addition, ISE2 also affects the interaction between the nematode and the host (Ganusova et al., 2017). *Potato leafroll virus* (PLRV), through the capsid readthrough domain (RTD), establishes extensive interaction networks with host chloroplast-localized proteins to increase its accumulation in host plants (Deblasio et al., 2018). RNAi, as the major antiviral mechanism of plants, can move between cells (Himber, 2003). BARELY ANY MERISTEM 1 (BAM1) and its closest homolog BAM2 in *Arabidopsis* play an important and redundant role in this process, and the C4 protein from tomato yellow leaf curl virus (TYLCV) interacts with BAM1 to inhibit the cell-to-cell spread of RNAi (Rosas-Diaz et al., 2018). *Bamboo mosaic virus* (BaMV) could regulate the relocalization of NbcpHsp70-2 in *N. benthamiana* to the chloroplasts in mature stage to enable its infection in mature tobacco leaves (Huang Y. W. et al., 2017). The sensitivity of tobacco to *tobacco mosaic virus* (TMV) depends on the leaf ages. TMV replicase can interact with the host phloem-related transcription factors in an age-dependent manner to regulate the transcriptional response to enhance plant phloem loading and the systemic spread of TMV in mature tissues (Collum et al., 2016). The TYLCV encoding a C4 protein relocalizes from the PM to the chloroplast upon the plant defense activation, interfering with the biosynthesis of SA. This pattern is observed in plant and several plant pathogens from different kingdoms. In plants, there may be a pathway that connects the PM to the chloroplast to activate the defense that is utilized by different pathogens to promote their infection by inhibiting the biosynthesis of SA and possible retrograde signaling (Medina-Puche et al., 2020).

## Concluding Remarks

**FIGURE 1:**
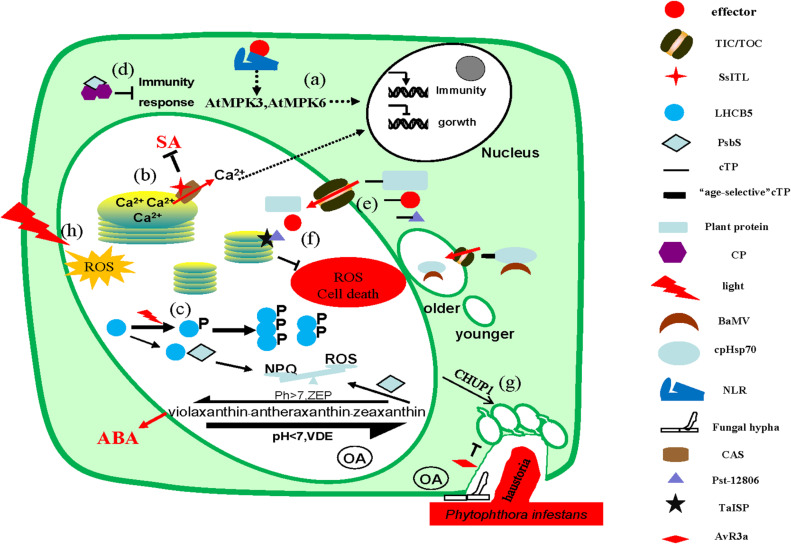
Microbes interfere with chloroplast functions. **(a)** Activated nucleotide-binding leucine-rich repeat domain-containing receptors (NLRs) activate MPK3 and MPK6 through unknown mechanisms, regulating the immune responses by increasing the expression of immune-related genes. **(b)** Once pathogen-associated molecular pattern (PAMP)-triggered immune signals are transported to the chloroplast, Ca^2+^ flows out from the thylakoid lumen to the stroma through the calcium-sensing (CAS) receptor, transmitting the signals to the nucleus via the retrograde signaling pathway, eliciting immune response. SsITL interacts with CAS to inhibit salicylic acid (SA) accumulation and promotes infection. **(c)** Phosphorylated LHCB5 exists as a dimer or trimer instead of a monomer, which weakens the interaction with PsbS and then weakens non-photochemical quenching (NPQ), promoting the production of reactive oxygen species (ROS). *S. sclerotiorum* secretes oxalate during infection to acidify the host tissue and accelerates the synthesis of zeaxanthin from violaxanthin, which reduced the production of ROS and ABA. **(d)** The CP protein of the virus interacts with PsbS as dimers in the cytoplasm to sequestrate PsbS from functioning. **(e)** Proteins enter the chloroplast by interacting with the translocator located in the inner membrane (TIC)/outer membrane (TOC) on the chloroplast membrane via chloroplast transit peptide (cTP). Moreover, the cTP of cpHsp70 protein is age selective and the *Bamboo mosaic virus* (BaMV) virus interacts with it to preferentially enter the mature chloroplasts for more energy. **(f)** Pst_12806 interacts with TaISP protein in chloroplasts to reduce ROS production and thus inhibit host cells death. **(g)** During the infection of *P. infestans*, CHUP1 anchors chloroplasts to the host–pathogen interface and restricts the entry of pathogen. Meanwhile, *P. infestans* secretes effector AVR3a to inhibit the formation of chloroplast stromules to reduce the contact area. **(h)** The production of ROS in chloroplast increases under light conditions.

The energy generated by chloroplasts through photosynthesis, including reactions like light absorption, electron transfer, photophosphorylation, and carbon assimilation, not only supports plant growth and development but also participates in plant defense responses. Besides, chloroplasts are also involved in plant immunity through the production of ROS, defense-related hormones, and retrograde signaling pathways. At the same time, chloroplasts are important signaling hubs that communicate with different cell compartments (Figure 1). Because of their important roles, chloroplasts are regarded as obstacles by various “enemies” of plants and is becoming an emerging battlefield in plant–pathogen interactions. Pathogenic microbes interfere with chloroplasts function and structure by delivering small molecules and effector proteins into the plant cells (Table 1). Correspondingly, plants actively exert the defense responses by recognizing the effectors and counteracting the functions of effectors. In the process of chloroplast and host–microbe interaction study, more interesting and fascinating functions of chloroplasts are slowly being elucidated, which has important implications for both plant growth and immunity.

## Author Contributions

FY wrote most part of this manuscript. KX helped to writing this manuscript. JL and HP supervised, revised, and complemented the writing.

## Conflict of Interest

The authors declare that the research was conducted in the absence of any commercial or financial relationships that could be construed as a potential conflict of interest.
